# The role of long intergenic non-protein coding RNA 312 in cancer: A bioinformatics and literature based study

**DOI:** 10.1016/j.bbrep.2025.102283

**Published:** 2025-09-25

**Authors:** Arash Safarzadeh, Setareh Ataei, Soudeh Ghafouri-Fard

**Affiliations:** Department of Medical Genetics, Shahid Beheshti University of Medical Sciences, Tehran, Iran

**Keywords:** LINC00312, Cancer, Bioinformatics

## Abstract

LINC00312 encodes an intronless transcript mainly functioning as a tumor suppressor. Expression of this transcript is downregulated in a variety of cancers, particularly lung cancers. It is regarded as a negative regulator of estrogen receptor signaling. Moreover, low expression of LINC00312 correlates with poor survival in sarcoma and stomach adenocarcinoma, suggesting its potential as a prognostic biomarker. In the current study, we performed a bioinformatics and literature based approach to find the importance of this long non-coding RNA in different cancers, its regulatory network and its interactions with different biomolecules and compounds. Taken together, LINC00312 represents a possible candidate for additional diagnostics and prognostics approaches.

## 1 Introduction

Long non-coding RNAs (lncRNAs) belong to a group of transcript with sizes more than 200 nt and wide involvement in the regulation of several biological and disease-related mechanisms within the cell at following levels: transcriptional, post-transcriptional, and epigenetic [1]. While they have no or limited coding capacity, they function at the epigenetic level through serving as scaffold of histone modification complexes and sponge for microRNAs [2]. Moreover, lncRNAs have pivotal roles in the commencement of reprogramming, preservation of pluripotency, and developmental processes through several *cis* and/or *trans* mechanisms [3]. Among these transcripts are long intervening/intergenic noncoding RNAs (lincRNAs) whose nucleotide sequences do not overlap protein-coding genes. LincRNAs may include promoter- or enhancer-associated sequences that are located in neighborhood of certain genes either in the sense or antisense direction, in relation with the corresponding gene [4]. They are involved in cancer progression through mechanisms such as chromatin remodeling, transcriptional regulation, and interaction with microRNAs (miRNAs) and proteins [5].

LINC00312 is an example of lincRNAs. This gene is located on 3p25.3 and produces an intronless transcript that mainly acts as a tumor suppressor. While it has shown differential expression in various cancers [6,7], data regarding the underlying mechanisms is obscure. Moreover, there is no comprehensive evaluation of the regulatory mechanisms, downstream pathways, the LINC00312/miRNA/mRNA axes and gene-disease and gene-drug networks of LINC00312. Advances in bioinformatics tools and high-throughput sequencing have facilitated the systematic exploration of function of this lincRNA, providing insights into its regulatory networks and clinical relevance. Thus, in this study, we used a combined bioinformatics approach and a comprehensive literature review to elucidate the role of LINC00312 in cancer. We analyzed its expression patterns across different cancer types, explored its interactions with protein-coding genes and signaling pathways, and assessed its prognostic and diagnostic potential. By merging current knowledge and leveraging computational predictions, this study aimed to provide a deeper understanding of LINC00312's functional significance in cancer biology, paving the way for future experimental validation and therapeutic targeting.

### 1.1 Basic information

**Fig. 1:**
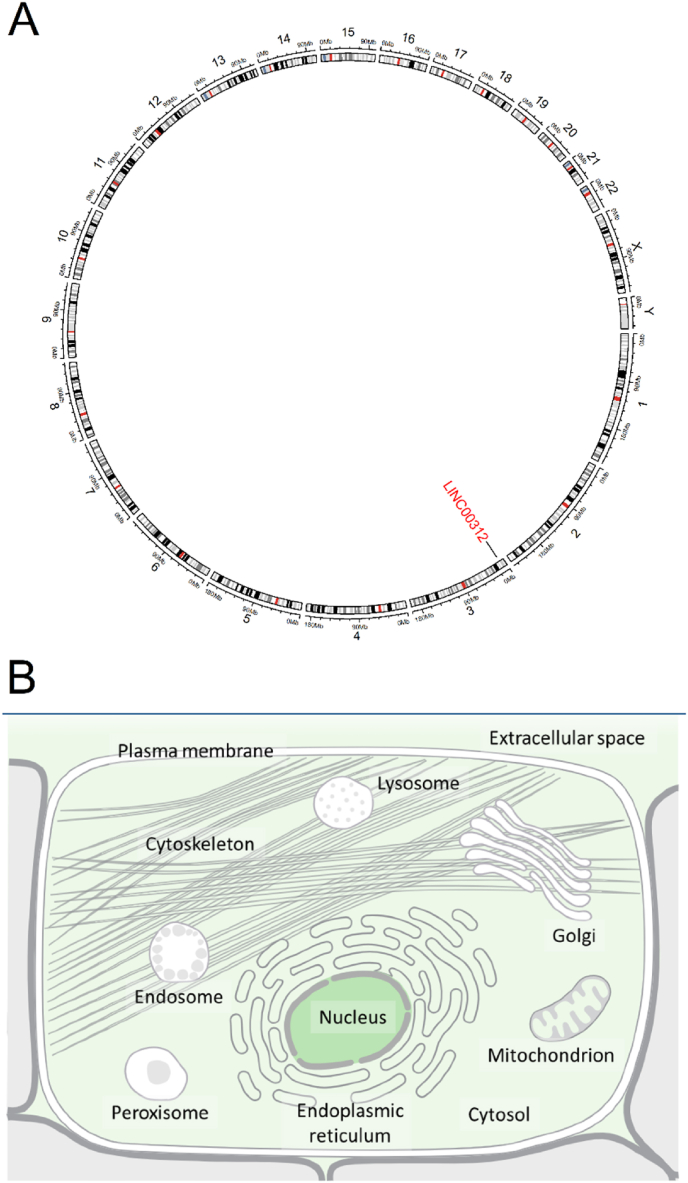
Chromosomal and cellular information of LINC00312. (A) Chromosomal location of LINC00312 displayed as a circular plot; (B) Subcellular localization of LINC00312.

Based on the Genomics for LINC00312 Gene information from the GeneCards database [8], and using the bioMart (version 2.58.2) and circlize (version 0.4.16) packages in R, we visualized the chromosomal location of LINC00312 as a circular plot (Fig. 1A). Also, based on the subcellular localization data, it has been determined that LINC00312 primarily functions in the cell nucleus (confidence score = 2) (Fig. 1B).

### 1.2 Survival analysis and Kaplan-Meier plots related to LINC00312

**Fig. 2:**
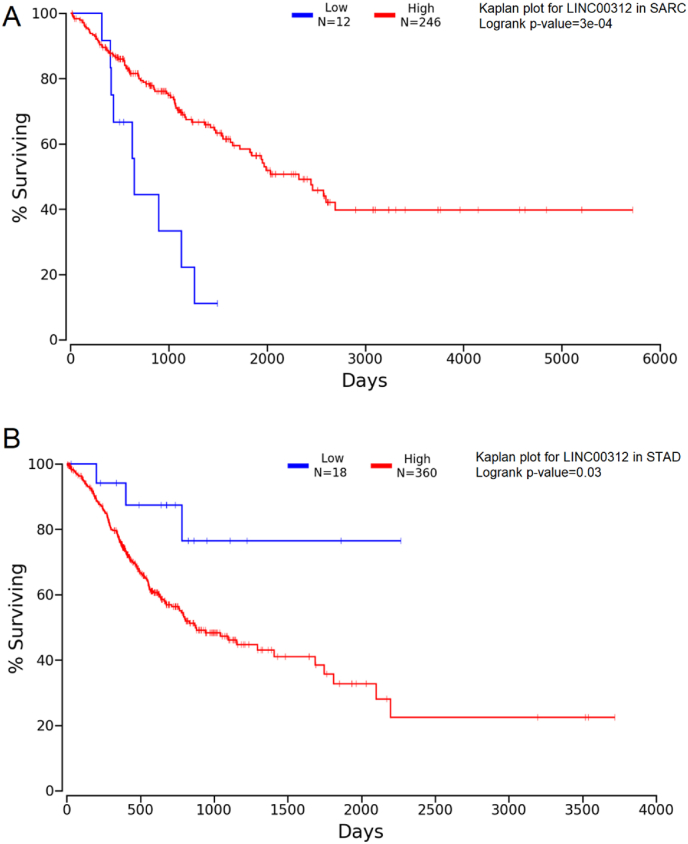
Pan-cancer survival analysis of LINC00312. (A) Kaplan–Meier survival plot in TCGA-SARC (sarcoma); (B) Kaplan–Meier survival plot in TCGA-STAD (stomach adenocarcinoma). Logrank P value < 0.05 was considered as significant.

Survival analysis via OncoLNC [9] and Kaplan-Meier plots demonstrated that LINC00312 significantly correlated (p < 0.05) with survival outcomes in sarcoma (TCGA-SARC) and stomach adenocarcinoma (TCGA-STAD) (Fig. 2A and B).

### 1.3 Genetic alterations in LINC00312

**Fig. 3:**
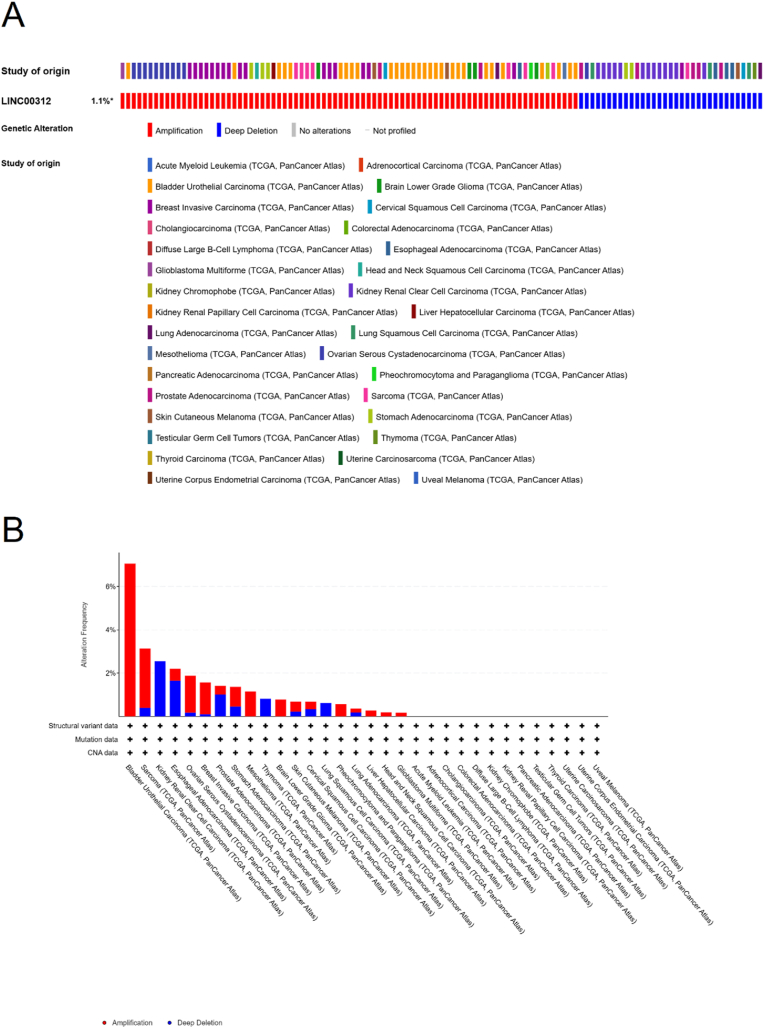
Pan-cancer mutation analysis of LINC00312. (A) Tumor samples harboring mutations in LINC00312; (B) Ratio of amplification to deep deletion mutations of LINC00312 across various cancer types.

Using the cBioPortal database [10] and searching for 'TCGA PanCancer Atlas,' we selected a total of 10,967 samples across 32 datasets to analyze LINC00312 mutations in a pan cancer analysis approach. Our analysis showed that LINC00312 was mutated in 1.1 % of the total samples, with 30 cases exhibiting amplification and 15 cases showing deep deletion (Fig. 3A). LINC00312 exhibited amplification mutations in samples from Bladder Urothelial Carcinoma, Mesothelioma, Brain Lower Grade Glioma, Pheochromocytoma and Paraganglioma, Liver Hepatocellular Carcinoma, Head and Neck Squamous Cell Carcinoma, and Glioblastoma Multiforme. Notably, more than 6 % of assessed Bladder Urothelial Carcinoma samples exhibited amplification of this lncRNA. Deep deletion mutations were found in samples from Kidney Renal Clear Cell Carcinoma, Thymoma, and Lung Squamous Cell Carcinoma. Meanwhile, a mixture of both amplification and deep deletion mutations was observed in samples from Sarcoma, Esophageal Adenocarcinoma, Ovarian Serous Cystadenocarcinoma, Breast Invasive Carcinoma, Prostate Adenocarcinoma, Stomach Adenocarcinoma, Skin Cutaneous Melanoma, Cervical Squamous Cell Carcinoma, and Lung Adenocarcinoma. Of note, about 3 % of Kidney Renal Clear Cell Carcinoma had deletions in this region (Fig. 3B).

### 1.4 LINC00312-target regulations in human cancers

Based on the LncTarD 2.0 database [11], we investigated the targets of LINC00312 as well as its regulatory roles in human cancers. According to the data from this database, LINC00312 plays a regulatory role in breast cancer, nasopharyngeal carcinoma (NPC), non-small cell lung cancer (NSCLC), oral submucous fibrosis, thyroid cancer, and urinary bladder cancer (Table 1).

**Table 1:** LINC00312-target regulations in human cancers.

Disease name	Regulator	Target	Influenced function	Regulatory mechanism
Breast cancer	LINC00312	CDH1	Cell proliferation (−), Colony formation (−), Cell migration (−), Cell invasion (−)	ceRNA(miR-9)
Nasopharynx carcinoma	LINC00312	PRKDC	MRN/ATM/CHK2 signaling pathway (−), ATR/CHK1 signaling pathway (−)	–
Non-small cell lung cancer	HOXA5	LINC00312	Cell proliferation (−), Apoptosis process (+)	transcriptional regulation
Oral submucous fibrosis	LINC00312	YBX1	Cell viability (+)	–
Thyroid cancer	LINC00312	PI3K	Cell proliferation (−), Cell invasion (−), PI3K/AKT signaling pathway (−)	–
Thyroid cancer	LINC00312	AKT1	Cell proliferation (−), Cell invasion (−), PI4K/AKT signaling pathway (−)	–
Thyroid cancer	LINC00312	miR-197-3p	Cell migration (−), Cell invasion (−)	–
Urinary bladder cancer	LINC00312	miR-197-3p	Cell migration (−), Cell invasion (−)	sponge

### 1.5 Identification of LINC00312 RNA interactions and lncRNA/miRNA/mRNA/TF and PPI networks construction

**Fig. 4:**
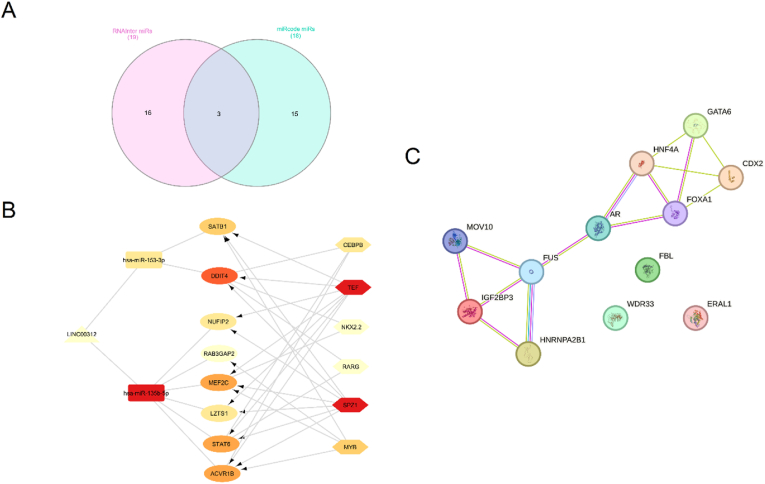
The ceRNA network related to LINC00312. (A) Venn diagram showing the intersection between miRNAs interacting with LINC00312 based on the miRcode database and those identified in the RNAInter database; (B) The ceRNA network; (C) The PPI network.

Based on RNAInter v4.0 (RNA Interactome Database) [12] LINC00312 has interactions with hsa-miR-197-3p, MOV10 and Temozolomide with score ≥0.3. Also, to identify interactions between LINC00312 and miRNAs, we utilized the RNAInter v4.0 and miRcode (http://www.mircode.org/) databases (Table S1). We then intersected the miRNAs obtained from these two databases, which ultimately revealed three miRNAs: hsa-miR-135b-5p, hsa-miR-153-3p, and hsa-miR-190a-5p (Fig. 4A). Subsequently, we employed the TargetScan, miRDB, and miRTarBase databases to obtain interactions between these miRNAs and mRNAs. Finally, we visualized the lncRNA/miRNA/mRNA interactions as a ceRNA network using Cytoscape software (version 3.10.3) [13]. Within this software, we used the iRegulon plugin [14] to identify transcription factors associated with the mRNAs, and based on the highest enrichment score for a transcription factor, we displayed the top 6 TFs in the network (Fig. 4B). Further investigations by the Harmonizome database [15] indicated that 14 transcription factors (CHD1, CTCF, EZH2, H2AFZ, HDAC6, JUN, KDM5A, MAFF, MAFK, PHF8, POLR2A, SAP30, SPI1 and TBL1XR1) possibly bind to the promoter of LINC00312 gene based on ChIP-seq data from the ENCODE Transcription Factor Target. Also, Using the LncSEA 2.0 database [16], we identified interactions between LINC00312 and 12 target mRNAs: AR, CDX2, ERAL1, FBL, FOXA1, FUS, GATA6, HNF4A, HNRNPA2B1, IGF2BP3, MOV10, and WDR33. Next, a protein-protein interaction (PPI) network was created using the STRING database [17] with a minimum required interaction score threshold of 0.4 (Fig. 4C).

### 1.6 Gene Ontology (GO) enrichment analysis of LINC00312-related MiRNAs

**Fig. 5:**
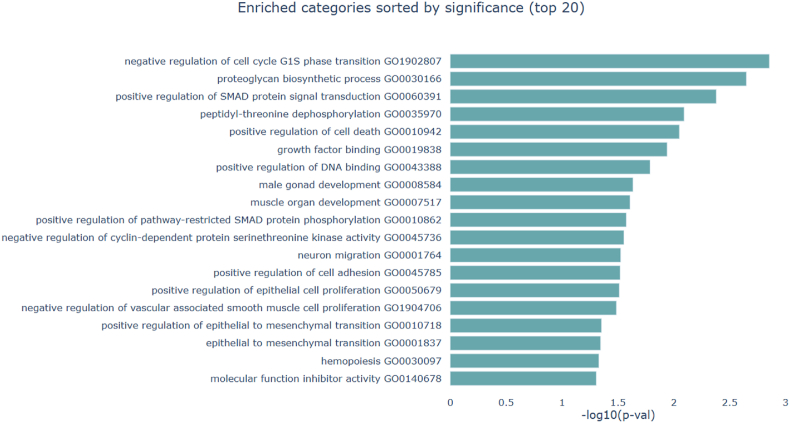
Gene ontology enrichment analysis of LINC00312-related miRNAs.

To perform GO enrichment analysis for the miRNAs associated with LINC00312 in the ceRNA network, we used the miEAA database [18]. Within this database, we applied the option "Annotations derived over miRTarBase (Gene Ontology)". As a result, a total of 193 GO terms with P < 0.05 were identified (Table S2). The top 10 enriched GO terms include: regulation of lung blood pressure (GO:0014916), semi-lunar valve development (GO:1905314), indoleamine 2,3-dioxygenase activity (GO:0033754), tryptophan 2,3-dioxygenase activity (GO:0004833), negative regulation of cell cycle G1/S phase transition (GO:1902807), endochondral bone morphogenesis (GO:0060350), negative regulation of chondrocyte proliferation (GO:1902731), proteoglycan biosynthetic process (GO:0030166), chondrocyte development (GO:0002063), and endocardial cushion development (GO:0003197) (Fig. 5).

#### 1.6.1 Gene-disease and gene-drug networks in LINC00312

**Fig. 6:**
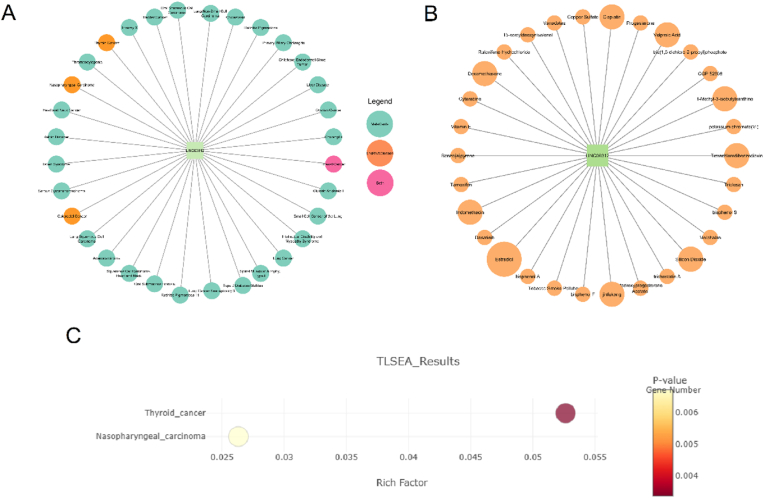
Association of LINC00312 with drugs and diseases. A) Gene–disease interaction network related to LINC00312; B) Gene–drug interaction network related to LINC00312; C) Dot plot representing enrichment analysis of LINC00312.

We identified diseases associated with LINC00312 using the MalaCards [19] and LncRNADisease v3.0 [20] databases (Table S3). According to the MalaCards database, NPC, Thyroid Cancer, Bladder Cancer, and NSCLC showed search score >10 with LINC00312. The LncRNADisease v3.0 database linked LINC00312 to Breast Neoplasms, Colorectal Neoplasms, Thyroid Neoplasms, and NPC. Notably, thyroid, colorectal, and nasopharyngeal cancers were consistently identified in both databases (Fig. 6A). Furthermore, according to the Comparative Toxicogenomics Database (CTD) [21], LINC00312 interacts with multiple compounds (counts ≥2), including: Estradiol, 1-Methyl-3-isobutylxanthine, Cisplatin, Dexamethasone, Indomethacin, Jinfukang, Silicon Dioxide, Tetrachlorodibenzodioxin, and Valproic Acid (Fig. 6B). The TLSEA database [22] analysis demonstrated a statistically significant association of LINC00312 with both thyroid cancer and NPC (P < 0.05), suggesting its potential functional involvement in these malignancies (Fig. 6C).

### 1.7 LINC00312 expression in cell lines

**Fig. 7:**
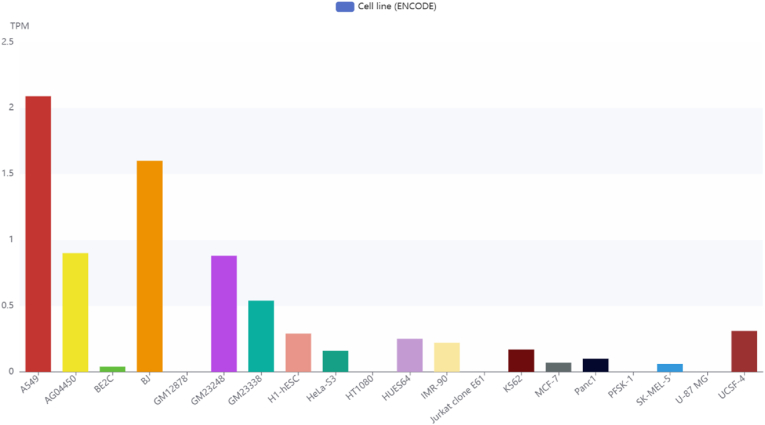
Expression levels of LINC00312 across different cell lines based on ENCODE data.

Using the LNCSEA 2.0 database and expression data from ENCODE cell lines, we explored the expression levels of LINC00312 across various cell lines. This lncRNA shows the highest expression in A549 (adenocarcinomic human alveolar basal epithelial cells), BJ (fibroblasts), and GM23248 (fibroblasts) cell lines (Fig. 7).

### 1.8 Literature search

Literature-based search showed down-regulation of LINC00312 in a variety of cancers (Table 2), including NSCLC [23], ovarian cancer [24], and head and neck cancers [25]. In some contexts (e.g., bladder cancer [26], lung adenocarcinoma (LUAD) [27]), it may be upregulated and function as an oncogene, promoting tumor progression. However, in these two cancer types, data regarding its expression pattern is conflicting.

**Table 2:** Expression of LINC00312 in different cancers.

Cancer Type	Expression/Role	Samples/Assessed Cell Lines	Pathways and Axes	Targets/Regulators	Function	Ref
Non-small cell lung cancer (NSCLC)	Downregulated/Tumor suppressor gene	319 patients and 180 healthy controls/BEAS-2B	–	–	A new non-invasive detection for NSCLC assessment was provided by the substantial decreased levels of circulating LINC00312 in NSCLC.	[23]
		92 paired tissues	–	–	In the initial phases NSCLC, 14 lncRNAs (HAGLR, ADAMTS9-AS2, LINC00261, MCM3AP-AS1, TP53TG1, C14orf132, LINC00968, LINC00312, TP73-AS1, LOC344887, LINC00673, SOX2-OT, AFAP1-AS1, and LOC730101) could distinguish between malignant and non-cancerous lung tissue, as well as LUAD and LUSC. A precise detection for NSCLC in its early stages may be possible with the use of the 14 lncRNA panel.	[28]
		100 paired tissues/A549, SPC‐A1, H1299, H1975, PC9, H1703, H520 and SK‐MES	HOXA5/LINC00312 axis	HOXA5	In NSCLC, LINC00312 is a new putative inhibitor of tumor growth that partially operates by preventing tumor formation and triggering apoptosis. HOXA5 might control LINC00312 expression.	[36]
		76 NSCLC tissues (43 LAD and 33 LSCC)/A549, SPC-A-1, H1299 95-D, HELF	–	–	LINC00312 may be a new marker for the initial recognition of lung cancer, which might be crucial for NSCLC therapy.	[37]
		57 tumor samples and 16 non-tumor samples	–	–	Comparing NSCLC tumors to normal lung tissues, LINC00312 is considerably down-regulated in the former. Such notable variations in expression might point to LINC00312's crucial regulatory role in the formation of NSCLC.	[38]
		Bioinformatics approach: GSE43458 dataset with 30 normal samples, 40 NSCLC tissues from never-smoking patients and 40 NSCLC tissues from smoking patients	–	–	Notably, the prognosis of patients with lung cancer was linked to a panel of genes that included DNAH7, DYNC2H1, WDR78, COL3A1, COL1A1, COL1A2, RXFP1, RAMP2-AS1, LINC00312 and LINC00472.	[31]
		Bioinformatics approach: 1525 samples from three data sets: GSE30219 (293), GSE31210 (226) TCGA (1006)	Ferroptosis signaling pathway	–	The nine ferroptosis-related lncRNAs (LINC00304, LINC00968, LINC00312, LINC00853, LINC-PINT, SLC12A2-DT, DANCR, LINC00491, and LINC00310) that make up the Risk-Score model can be a great way to predict prognosis and the response to immunotherapy for NSCLC.	[32]
Ovarian high-grade serous carcinoma (Ovarian HGSC)	Downregulated/Tumor suppressor gene	22 HGSC tissue samples, 10 normal ovarian tissues and 10 normal fallopian tube tissues/CaOV3, OVCAR3	–	–	This study identified 11 downregulated lncRNAs, including LINC00312, in patients with high-grade serous carcinoma (HGSC).	[24]
Ovarian cancer (OC)	Downregulated/Tumor suppressor gene	60 chemosensitive patients and 60 chemoresistant patients/SKOV3, SKOV3/DDP	Bcl-2/Caspase-3 signaling pathway	miR-197-3p	Through the Bcl-2/Caspase-3 signaling pathway, LINC00312 induced cell death, boosting the susceptibility of SKOV3/DDP cells to cisplatin. According to these results, LINC0312 may represent an advantageous therapeutic approach for treating ovarian cancer that is resistant to drugs.	[33]
Oral squamous cell carcinoma (OSCC)	–	469 male patients with squamous cell carcinoma of buccal mucosa and 1194 cancer-free males	–	–	There is a relationship between the incidence of nodal spread in buccal mucosal carcinoma and LINC00312 rs164966. Only individuals who regularly smoked cigarettes or used betel quid had this genetic link, while those who were not exposed to these serious environmental hazards did not have it. The metastatic potential of oral cancer may be impacted by LINC00312 polymorphisms in combination with repetitive exposure to carcinogens resulting from lifestyle-related hazards.	[39]
Head and neck cancer (HNC)	Downregulated/Tumor suppressor gene	OECM1, SAS, FaDu, Detroit 562 (Detroit), and CGHNC8	–	–	This work revealed LINC00312 as a critical component of the lncRNA profiles associated in areca nut-induced HNC. The development of targeted therapies, early diagnosis, prognosis assessment, and risk stratification for areca nut-associated cancers are among the clinical applications of these molecular markers, especially LINC00312-mediated regulatory networks.	[25]
Nasopharyngeal carcinoma (NPC)	Downregulated/Tumor suppressor gene	505 NPC patients	–	–	LINC00312 genetic variants may be used as biomarkers for personalized treatment and influence the risk of chemoradiotherapy-induced hematotoxicity in patients with NPC.	[30]
		232 Non-cancerous epithelial and 329 NPC samples	–	–	The multi-step development of human nasopharyngeal epithelial carcinogenesis may include LINC00312. One important independent predictor of the transition from non-cancerous epithelium to NPC is LINC00312.	[40]
		92 NPC tissues and 10 chronic inflammation of nasopharyngeal mucosa tissues/NP69, CNE1, CNE2, HNE1, and HONE1	AKT–DNA–PKcs axis/NHEJ repair pathway	DNA-PKcs	LINC00312 attaches itself to DNA-PKcs and prevents it from being recruited to Ku-DNA. Inhibiting irradiation-induced AKT–DNA–PKcs, MRN–ATM–CHK2, and ATR–CHK1 signal transduction, LINC00312 impairs the ability to detect, process, and repair DNA damage and makes a person more susceptible to radiation treatments.	[6]
		684 histopathology-confirmed NPC patients	–	–	The SNPs of LINC00312 were linked to survival and susceptibility to NPC, maybe through affecting linc00312 expression.	[41]
Bladder cancer (BC)	Downregulated/Tumor suppressor gene	110 specimens from 110 patients/T24, BIU87, 5637 and SV-HUC-1	–	miR-197-3p	BC cells with upregulated LINC00312 or downregulated miR-197-3p displayed high TIMP2 but low MMP-2 and MMP-9 amounts. By modulating miR-197-3p, LINC00312 prevented BC cell invasion and metastasis.	[34]
	Upregulated/Oncogene	52 paired tissues /T24, SW780 and HUM-CELL-0046	PI3K/AKT signaling pathways	–	Relative with healthy tissues, bladder cancer tissues had a greater amount of LINC00346. Bladder cancer cell migration and growth were decreased by LINC00346 elimination, which led to cell cycle arrest and cell death.	[26]
Lung adenocarcinoma (LUAD)	Downregulated/Tumor suppressor gene	6 stage I LUAD tissues and 6 adjacent non-tumor tissues	Cell adhesion molecules, focal adhesion, tight junction, pathways, angiogenesis and regulation of cell proliferation and apoptosis	–	Based on The Cancer Genome Atlas database, LINC00312 and FENDRR are useful for diagnosing LUAD patients. The pathophysiology of LUAD may be clarified by this work, and new biomarkers for patient diagnosis and possible therapeutic options may be established.	[29]
	Upregulated/Oncogene	124 paired tissues/H1703, H2009, PC-9, H1299, A549, and H1975	–	YBX1	LINC00312 directly binds to YBX1 to cause vasculogenic mimicry (VM) and lung cancer metastases. This work represents a major breakthrough in our current knowledge of LINC00312's multiple functions in lung cancer.	[27]
	Downregulated/Tumor suppressor gene	–	LINC00312/miR-3175/SEMA6A axis	miR-3175/SEMA6A	The activity of LINC00312 on lung cancer cells may be hampered by inducing miR-3175 or by suppressing SEMA6A. LINC00312 blocks the occurrence of lung cancer via the miR-3175/SEMA6A axis.	[35]
Hepatocellular carcinoma (HCC)	Downregulated/Tumor suppressor gene	23 pairs of HCC tissues and 75 adjacent normal liver tissues/HepG2	Apoptosis activation and cell cycle arrest	Cyclin B1	LINC00312 limits the growth of human HCC cells. One of Lnc00312's primary target proteins in human HCC cells may be cyclin B1.	[42]
Colorectal cancer (CRC)	Downregulated/Tumor suppressor gene	22 CRC specimens and their matched adjacent normal tissues/SW480, SW620, LoVo and HT29	miR-21/PTEN axis	miR-21/PTEN	The LINC00312-miR-21-PTEN axis might promote the growth of CRC cells and the creation of tumors, and it may result in the development of novel lncRNA-based CRC diagnostics or treatments.	[43]
Breast cancer (BC)	Downregulated/Tumor suppressor gene	26 paired tissues/HMECs, MCF-7, T47D, BT549, MDA-MB-231 and SKBR3	miR-9/CDH1 axis	miR-9/CDH1	The downregulation of VIM and overexpression of CDH1 caused by LINC00312 were partially reversed by miR-9. The LINC00312/miR-9/CDH1 axis may thus play a part in the development of BC, which implies a new lncRNA-based diagnostic biomarker or potential treatment target for BC.	[7]
Glioma	Downregulated/Tumor suppressor gene	35 patients with glioma	–	miR-21-3p	As a molecular sponge, LINC00312 could attract miR-21-3p, controlling glioma cell invasion, growth, and EMT and contributing to glioma cell inhibition.	[44]
Pancreatic cancer (PC)	Downregulated/Tumor suppressor gene	–	–	FGD4	By adversely controlling FGD4, LINC00312 overexpression limits PC cell migration and growth. LINC00312 is therefore a possible target for pancreatic cancer treatment.	[45]
Epstein–Barr virus-related cancer	–	–	–	–	EBV-encoded factors directly and indirectly modulate host lncRNAs (e.g., LINC00312) through diverse mechanisms to promote oncogenesis.	[46]

LINC00312 is part of multi-lncRNA panels for early cancer detection (e.g., NSCLC [28] and LUAD [29]). Moreover, its expression correlates with patient survival (e.g., in NPC [30], NSCLC [31]).

LINC00312 is involved in the regulation of PI3K/AKT pathway (bladder cancer) [26], ferroptosis (NSCLC) [32], and apoptosis (ovarian cancer) [33]. Moreover, it binds to a variety of miRNAs (e.g., miR-197-3p in bladder cancer [34], and miR-3175 in lung cancer [35]) to modulate gene expression. It is also involved in DNA repair mechanisms. In NPC, LINC00312 disrupts DNA-PKcs recruitment, sensitizing cells to radiation [6].

## 2 Discussion

The complete analysis of LINC00312 across various cancer types, as presented in this article, reveals its multifaceted role in oncogenesis, tumor suppression, and clinical utility. Kaplan-Meier plots demonstrated that low expression of LINC00312 correlates with poor survival in sarcoma and stomach adenocarcinoma, suggesting its potential as a prognostic biomarker. This aligns with its tumor-suppressive role in other cancers, such as NSCLC [23], ovarian cancer [24], and head and neck cancers [25], where its downregulation promoted aggressive phenotype.

The correlation between genomic alterations in LINC00312 and carcinogenesis is unclear. While it exhibited amplifications in some aggressive cancers (e.g., glioblastoma, HCC), deep deletions were observed in some others (e.g., kidney renal cell carcinoma), indicating context-dependent roles. The mixed alterations in cancers like LUAD and breast cancer further underscore its dual functionality as either an oncogene or tumor suppressor.

From a mechanistical viewpoint, LINC00312 mainly acts through ceRNA mechanism, For instance, in breast cancer, LINC00312 sponges miR-9 to upregulate CDH1 [7], inhibiting metastasis. It also suppresses PI3K/AKT in thyroid cancer and regulates Bcl-2/Caspase-3 in ovarian cancer, promoting apoptosis. However, its interaction with YBX1 in LUAD drives vasculogenic mimicry [27], highlighting its pro-metastatic role in certain contexts. Three miRNAs, namely hsa-miR-135b-5p, hsa-miR-153-3p, and hsa-miR-190a-5p have been shown to interact with LINC00312. miR-135b-5p has been recently shown to promote gastric carcinogenesis through modulating CLIP4-mediated JAK2/STAT3 signaling [47]. miR-153 is principally recognized miRNA that regarded as a tumor suppressive miRNA regulating genes associated with cancer development [48]. Finally, abnormal expression of miR-190 has been reported in several types of cancers. It contributes to numerous cancer-related biological processes, such as proliferation, apoptosis, metastasis and drug resistance [49].

LINC00312 has interactions with several signaling pathways ferroptosis, Bcl-2/Caspase-3 AKT–DNA–PKcs axis, NHEJ repair pathway, and PI3K/AKT signaling pathways.

LINC00312 interactions with **cisplatin**, **estradiol**, and **dexamethasone**, as revealed through assessment of Gene-Drug Networks suggest its role modulation of chemosensitivity. The role of this lincRNA in enhancement of cisplatin efficacy has also been confirmed in ovarian cancer [33].

## 3 Conclusions

In brief, LINC00312 is a lincRNA with context-dependent roles in cancer, functioning as either a tumor suppressor or oncogene. The conflicting expression patterns of LINC00312 in certain cancers may show its tissue-specific functions or distinct cellular processes affected by this lncRNA in certain contexts. Its involvement in critical pathways, diagnostic potential, and therapeutic relevance highlight its importance in cancer research. Moreover, it may be a therapeutic target, for instance for drug-resistant cancers (e.g., cisplatin-resistant ovarian cancer). Further studies are needed to explore its mechanisms and translational applications. Moreover, the dual oncogenic/tumor-suppressive functions of LINC00312 should be considered in design of any targeted therapy. This also highlights the necessity for additional mechanistic studies to delineate tissue-specific regulators. Finally, while our *in silico* analyses showed strong correlations between LINC00312 and both survival and drug response, experimental validation in preclinical models is necessary to advance therapeutic strategies.

## CRediT authorship contribution statement

**Arash Safarzadeh:** Formal analysis, Data curation, Conceptualization. **Setareh Ataei:** Formal analysis, Data curation. **Soudeh Ghafouri-Fard:** Writing – review & editing, Writing – original draft, Supervision.

## Ethics approval and consent to participant

Not applicable.

## Consent of publication

Not applicable.

## Availability of data and materials

All data generated or analyzed during this study are included in this published article and its supplementary files.

## Funding

Not applicable.

## Declaration of competing interest

Authors declare no conflicts of interests.

## Data Availability

No data was used for the research described in the article
